# Insights into Biomarkers of Alzheimer's Disease: From Core Markers to Emerging Directions

**DOI:** 10.14336/AD.2025.0761

**Published:** 2026-07-17

**Authors:** Weiyao Zhu, Yu Wang, Ming Qin, Qinghe Zhao, Lina Feng, Mingquan Li

**Affiliations:** ^1^College of Traditional Chinese Medicine, Changchun University of Chinese Medicine, Changchun, 130117, China.; ^2^Department of Neurology, the Third Affiliated Clinical Hospital of the Changchun University of Chinese Medicine, Changchun, 130117, China.; ^3^Institute of Chinese Materia Medica, China Academy of Chinese Medical Sciences, Beijing, 100700, China.; ^4^Department of Neurology, the Second Affiliated Hospital of Shandong First Medical University, Shandong First Medical University & Shandong Academy of Medical Sciences, Taian, China.

**Keywords:** Alzheimer's disease, biomarkers, csf, body fluid, blood, review

## Abstract

Alzheimer's disease (AD) represents a neurodegenerative condition characterized by steadily increasing prevalence and incidence, arising significant challenge to both patients and social insurance. However, the etiology of AD remains controversial so far, and pathogenesis is far more complicated. Presently, no definitive therapeutic methodologies were available for AD, and only partial symptomatic relief can be achieved. Consequently, early diagnosis and intervention are emergently needed for AD patients. The diagnostic criteria for AD are continuously evolving, and biomarker testing is becoming increasingly critical for diagnosis. Currently, the diagnosis of AD primarily relies on the detection of pathological proteins through cerebrospinal fluid (CSF) testing and positron emission tomography (PET). However, factors such as high costs, operational contraindications, and invasiveness limited the application of these technologies, making them particularly challenging to implement in large-scale clinical trials and screenings. Core fluid biomarkers for AD including β-amyloid (Aβ), phosphorylated tau protein (p-tau), total tau protein (t-tau), and their combinations were found in CSF. Although these biomarkers were demonstrated with significant specificity and sensitivity, challenges remain high concerning the collection of CSF. Blood-derived biomarkers for Aβ and tau proteins are essential for preliminary screening, diagnosis, and monitoring of AD. Additionally, other bodily fluids such as saliva, urine, and tears have been investigated for their potential as biomarkers, offering unique characteristics and applications. Emerging biomarkers, including neurofilament light chain (NfL), neurogranin (Ng), Beta-site APP cleaving enzyme 1 (BACE1), synaptosome associated protein 25 (SNAP-25), as well as inflammation-related and gene-related factors, provided valuable insights into the diagnosis and pathogenesis of AD from diverse perspectives. Despite the substantial progress made in AD biomarker research, there are still baskets of limitations concerning the complication of the disease. The current review focused on the reported literature to summarize the biomarkers associated with AD. By critically analyzing studies published over the past decade, we aimed to strengthen the recent research progress, theoretical frameworks, and unresolved challenges related to AD biomarkers.

## 1. Introduction

Alzheimer's disease (AD) and other cognitive disorders constituted a group of neurodegenerative diseases that were typically manifested in the elderly and pre-elderly populations. These conditions were characterized by insidious onset and progressively deteriorating processes. The primary clinical manifestation is acquired cognitive impairment, affecting various cognitive domains, including memory, learning, orientation, comprehension, judgment, calculation, language, and visuospatial abilities. These impairments, to some extent, had impact on daily living skills and social occupational functions, and ultimately progressed to full-blown dementia [1]. Among dementia patients, AD is the most prevalent type, accounting for approximately 65% of all cases [2]. According to the latest international data, the prevalence of AD in Europe is projected to double by the year 2050, indicating a global increase of two-fold [3]. Currently, AD ranks among the most costly, lethal, and burdensome illnesses of this century [4]. This situation posed significant challenge to the well-being of elderly individuals and the happiness of their families, exerting considerable stress on both communities and households. It represents a major issue for societies experiencing aging populations.

The unusual accumulation of extracellular β-amyloid (Aβ) along with the development of neurofibrillary tangles (NFTs), which arise due to the hyperphosphorylation of the intracellular microtubule-associated protein Tau (MAPT), constitutes two essential pathological characteristics of AD [3]. Research has demonstrated that varying degrees of Aβ deposition and elevated levels of Tau protein were presented in the brains of individuals prior to the manifestation of symptoms associated with AD [5]. This finding suggested that the pathological changes associated with AD occurred during the early preclinical stage. However, the initial symptoms of AD are often atypical and challenging to detect, making early diagnosis difficult. Consequently, by the time the clinical symptoms were manifested, the optimal window for intervention and treatment would be frequently missed. Identifying the biomarkers that can effectively detect AD in its early phases is thus of great significance. In July 2024, the Alzheimer's Association, along with the National Institute on Aging, introduced updated criteria for diagnosing and staging Alzheimer's disease (2024) [6]. This revision built upon the ATN framework, which is based on biomarkers for Aβ (A), tau pathology (T), and neurodegeneration (N), by including additional non-specific biomarkers related to the development of AD (ATXN). Among the different approaches to identifying 'ATN' biomarkers, positron emission tomography (PET) and tests of cerebrospinal fluid are acknowledged as the most recognized protocols. However, their application has been limited by several factors. In contrast, peripheral blood testing might offer a more promising alternative for widespread application due to their lower cost and greater accessibility. Recent advancements in detection technologies have also enabled the identification of additional biomarkers. This review systematically synthesized and summarized the as-reported references on biomarkers associated with AD. Through a critical analysis of research published over the last decade, this overview aimed to provide a theoretical foundation for the development of more accurate and convenient methods for the early diagnosis of AD.

## 2. Search Strategy

We conducted a comprehensive search across various databases, including Google Scholar, PubMed, the Chinese National Knowledge Infrastructure database, the Chinese Medical Journal full-text database (yiigle.com), and the Excerpta Medica (EMBASE) database. The search utilized the following keywords: 'Alzheimer’s disease,' 'AD,' 'Biomarker,' 'Biological marker,' 'Biologic marker,' 'Markers biological,' 'Immunologic markers,' 'Serum markers,' 'Clinical markers,' 'Biochemical marker,' and 'Surrogate marker,' covering the period from 2015 to 2025.

## 3. Diagnosis and staging of AD

The development of AD was associated with intricate mechanisms, with primary pathological theories focusing on the accumulation of Aβ, the creation of NFTs, and the degeneration of neurons [7]. In individuals affected by AD, a particular form of protein builds up, referred to as "plaques" and "tangles." These proteins play a significant role in the deterioration of neural connections, which ultimately results in the death of neurons and a decrease in brain tissue [8]. A definitive diagnosis of AD is established through histopathological evidence of Aβ and phosphorylated tau (p-tau) proteins observed in the brain post-mortem [9]. The diagnostic criteria for AD have continuously evolved with newer investigations, transitioning from a purely pathological diagnosis in the early era of AD to the clinical exclusion method established by the Alzheimer's Disease Association (NINCDS-ADRDA) in 1984. This method was based on patient complaints, cognitive assessments, and imaging techniques, but did not include biomarkers. In 2007, the International Working Group (IWG) updated the criteria to incorporate biomarkers like Magnetic Resonance Imaging (MRI), PET scans, and analysis of cerebrospinal fluid (CSF). In 2011, the NINCDS-ADRDA introduced dual criteria, expanding the diagnostic scope and clarifying the role of biomarkers in diagnosing the various stages of the disease. In 2014, the IWG revised the criteria once again, emphasizing that biomarkers are essential for diagnosis, with equal importance placed on clinical symptoms and biomarkers [10].

**Figure 1.**
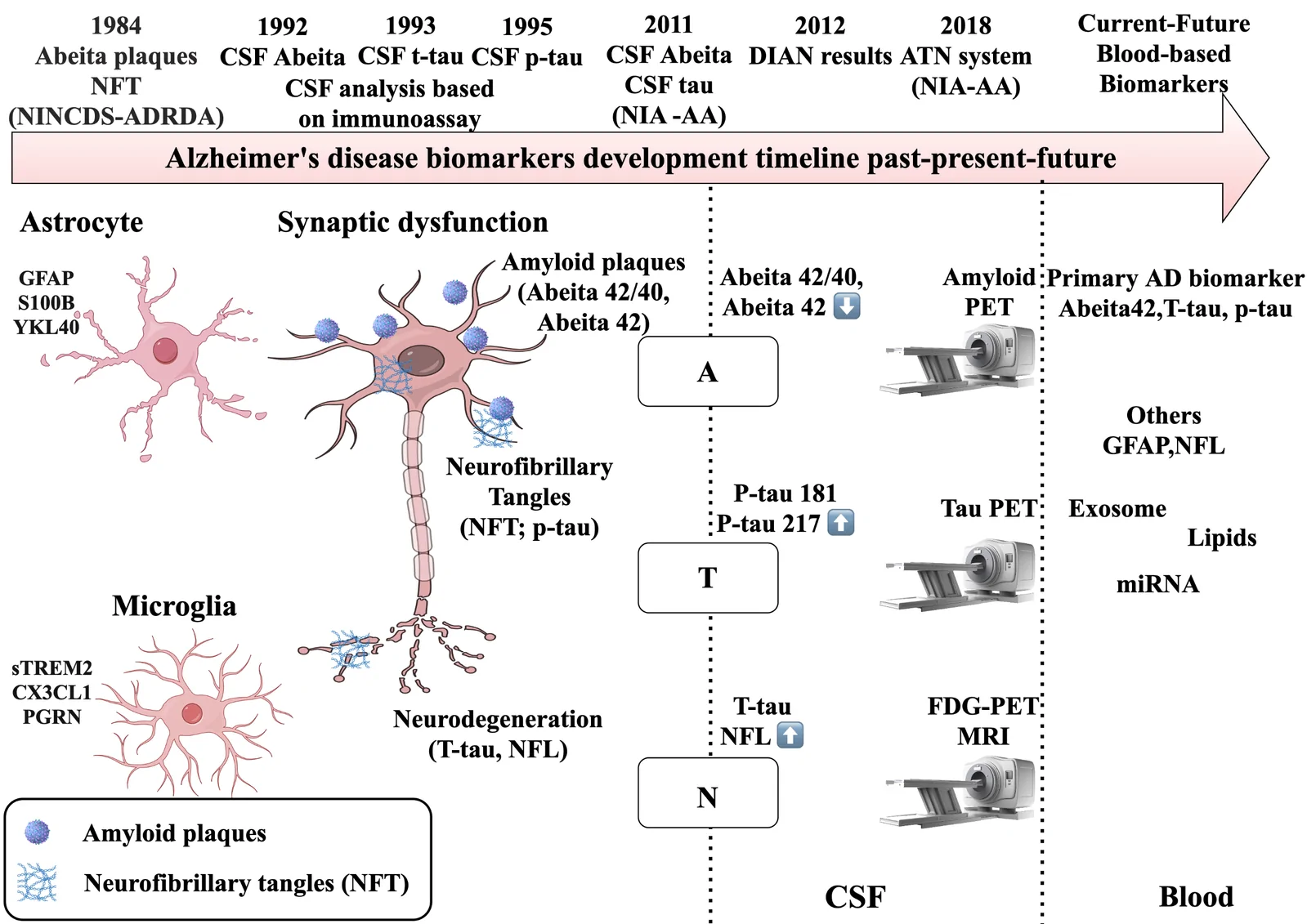
**The milestones in the Evolution of Alzheimer's Disease Biomarkers**. In 1984, the initial criteria for the clinical diagnosis of AD, known as the NINCDS-ADRDA criteria, were published. A definitive diagnosis of AD can only be confirmed through the detection of Aβ plaques and NFT via autopsy. Subsequently, brain imaging technologies capable of detecting amyloid accumulation in the brain, utilizing various PET ligands that bind to amyloid plaques, have emerged; more recently, tau PET imaging has been introduced. In 2011, the NIA-AA recommended diagnostic guidelines for both asymptomatic (i.e., preclinical) and symptomatic stages. The updated NIA-AA guidelines in 2018 revised the diagnostic criteria for research purposes and introduced a new diagnostic approach based on the ATN classification system within the research framework. In 2024, the application of newly added AD biomarkers in diagnosis includes Core 1: CSF Aβ42/40, CSF p-tau181/Aβ42, CSF t-tau/Aβ42, plasma p-tau217, plasma p-tau217/n-p-tau217, and Aβ PET. A positive result in Core 1 fulfills the biological diagnosis of AD. Core 2 involves tau PET, where a positive result completes the staging of AD biomarkers.

In 2018, the National Institute on Aging and the Alzheimer's Association (NIA-AA) proposed a biological definition of AD through the ATN diagnostic criteria [11]. The presence of biomarkers (A) alone is referred to as "AD pathological change," whereas the coexistence of biomarkers (A) and (T) is defined as AD, with (N) serving solely as an auxiliary indicator for assessing the progression or severity of AD pathology. This diagnostic criterion has been widely adopted and recognized in clinical practice. Clinically, methods for obtaining evidence of ATN include CSF measurement, MRI, PET, as well as the recent advancements in PET/CT and PET/MR imaging. Based on the updated guidelines for diagnosing and staging AD in 2024, it is essential to identify abnormalities in key biomarkers for an accurate diagnosis. These biomarkers included Aβ PET in molecular imaging, plasma p-tau217, and the ratio of p-tau217 to non-p-tau217 in fluid biomarkers, along with CSF p-tau181/Aβ 42, t-tau/Aβ 42, and Aβ 42/40. The identification of one or more positive indicators among these six markers is consistent with the biological diagnosis of AD. The evolution of AD biomarkers was shown in Figure 1.

## 4. Current Status of AD Treatment

The treatment of AD continues to present significant challenges, particularly given its rising incidence rate. Currently, no ultimate therapeutic methods are available [12]. Regarding the pharmacological treatment, only two categories of medications have received approval: cholinesterase inhibitors (such as donepezil and rivastigmine) and memantine, which modulates glutamatergic signaling by inhibition of the N-methyl-D-aspartate (NMDA) receptors [13]. In November 2019, the National Medical Products Administration of China approved GV-971, a medication aimed at the brain-gut axis for AD, for those suffering from mild to moderate forms of the condition [14]. In June 2021, Aducanumab, a monoclonal antibody belonging to the human immunoglobulin gamma 1 (IgG1) class and designed for the treatment of AD, obtained its first approval in the United States for patients exhibiting mild cognitive impairment (MCI) or those in the initial phases of mild dementia [15]. In January 2024, the injection of Lecanemab received approval for treating MCI and MCI associated with AD. Additionally, the non-pharmacological interventions primarily focus on cognitive training, lifestyle modifications, and psychological support.

In recent years, several emerging therapeutic strategies have been developed, including gene therapy, which can modulate neuroinflammation and shows showed significant promise for treating neuro-degenerative disorders [16]. Neuroinflammation plays a crucial role in the development of AD [17]. The inappropriate activation of astrocytes and microglia can damage neurons and facilitate the spread of tau pathology. Single-cell transcriptomics have uncovered the heterogeneity and functional diversity of glial cell subtypes in AD [18, 19]. The presence of different forms of the triggering receptor expressed on myeloid cells 2 (TREM2) in microglial cells is linked to a higher likelihood of developing AD [20]. TREM2 agonists can enhance microglial activation and their aggregation around Aβ, therefore reducing neuroinflammation and increasing the dissemination of tau pathology [21]. In addition, research indicated that that passive immunotherapy with monoclonal antibodies targeting Aβ is a promising therapeutic strategy for AD. The drainage of lymphatic fluid in the meninges plays a key role in the accumulation of Aβ in the brain, and its efficiency significantly influences the effectiveness of immunotherapy for AD. Diminished drainage of meningeal lymphatics can worsen inflammatory reactions in microglia, while improving the function of meningeal lymphatics alongside immunotherapy might lead to better treatment results [22]. Meningeal lymphatic vessels (mLVs) have been implicated in the clearance of Aβ, indicating that they may be potential therapeutic targets for AD. Based on this foundation, therapy utilizing near-infrared light has been shown to enhance the capacity of meningeal lymphatic vessels to clear Aβ and improve the cognitive functions in both aged and AD mice, thereby presenting a promising treatment avenue for neuro-degenerative disorders [23].

The approval of Aβ-targeting monoclonal antibody drugs has instilled hope for the treatment of AD [24]. However, their clinical application necessitates precise diagnostic measures. In this context, the screening, early diagnosis, and treatment of AD at the preclinical stage are of paramount importance. Consequently, the identification of accurate, convenient, and cost-effective biomarkers is becoming increasingly crucial in the clinical practice of AD management.

## 5. Updated Classification of AD Biomarkers

Before the 21st century, AD could be diagnosed solely through examinations of brain pathology after death. By the beginning of the 21st century, the identification of AD pathology in living patients was made possible through the use of biomarkers found in CSF and PET imaging [25]. In 2018, the NIA-AA proposed the AT (N) research framework aimed at diagnosing AD, providing a biological definition of the disease [11], and marking the transition of AD diagnosis into the era of biomarker precision. To the best of our knowledge for AD pathology, ATN is evolving into ATX (N), where 'X' denotes additional pathophysiological markers such as inflammatory or vascular factors. The revised criteria for diagnosis and staging of AD in 2024 categorized biomarkers into three major types: core AD biomarkers, non-specific AD biomarkers, and non-AD comorbid pathological biomarkers. Furthermore, it introduced the 'ATNIVS' biomarker framework, which incorporates 'I' to represent inflammatory mechanisms, as well as two common types of non-AD comorbidities: vascular brain damage (V) and alpha-synuclein (S). The expanded ATNIVS framework, which encompassed inflammation, vascular injury, and alpha-synuclein, offered more comprehensive diagnostic coverage.

**Figure 2.**
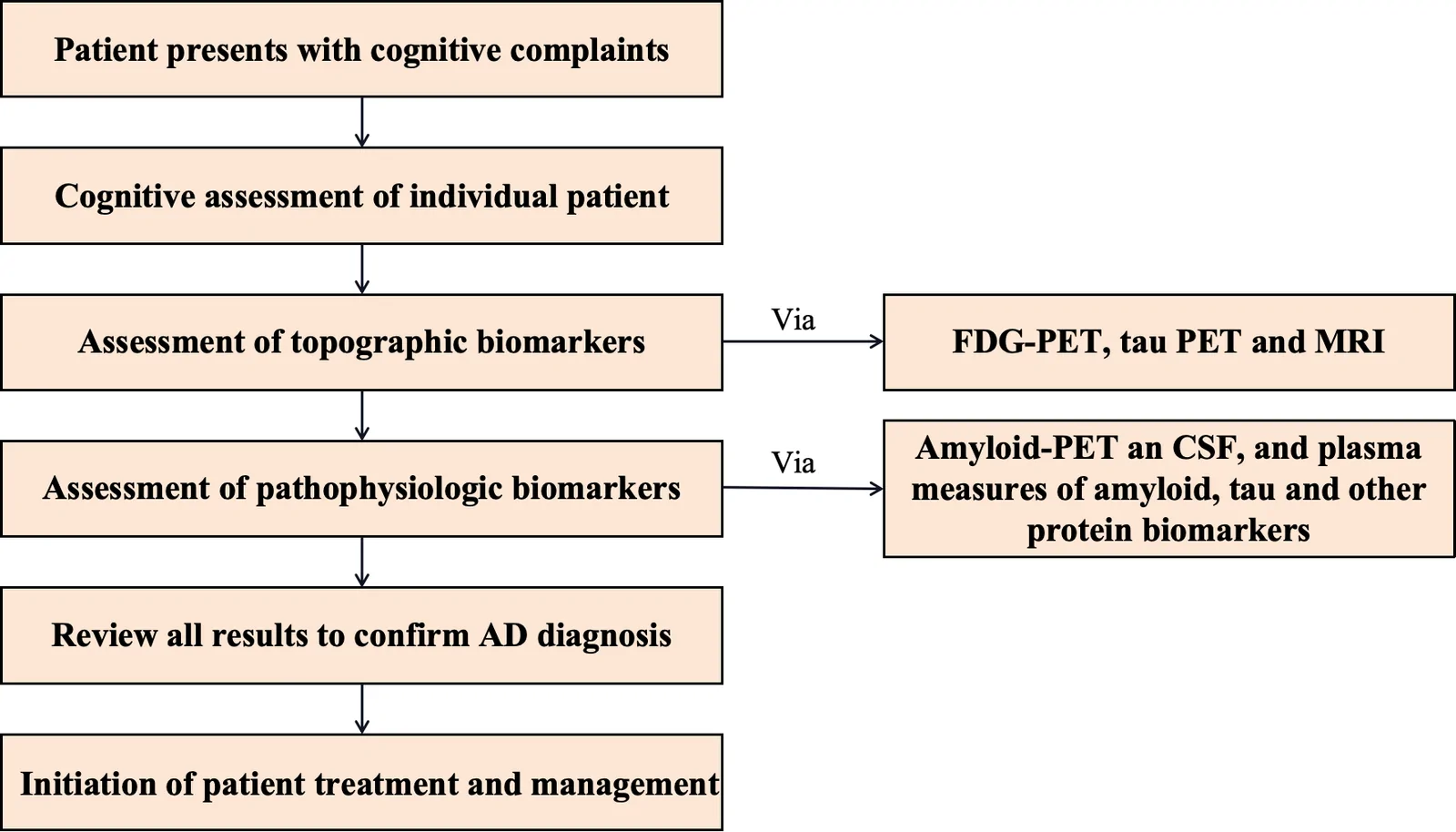
The role of AD biomarkers in the clinical setting [168].

The updated standards have been revised based on the 2018 research framework document by incorporating plasma biomarkers as core indicators for the qualitative diagnosis of AD. These biomarkers, collectively referred to as fluid biomarkers, are used along with CSF markers for both qualitative diagnosis and biomarker staging of AD. Non-specific biomarkers for AD are crucial in understanding the pathological progression of the disease. The designation "N" represents neuronal injury or neurodegeneration, with the inclusion of NfL in the new standards indicating axonal damage in neurons, which can be observed in both CSF and plasma. Neuronal and synaptic damage may also lead to decreased brain gray matter volume (or cortical thickness) and reduced fluorodeoxyglucose (FDG) metabolism; therefore, structural MRI and FDG-PET serve as imaging biomarkers for "N." The designation "I" represents inflammation, with the newly included glial fibrillary acidic protein (GFAP) serving as an indicator of astrocyte activation, detectable in both plasma and CSF. Among the pathological markers of non-AD comorbidities, "V" denotes the vascular brain injury, while "S" refers to alpha-synuclein. Although "V" and "S" cannot be utilized for the qualitative diagnosis and biomarker staging of AD, they frequently coexist with AD and can assist in differential diagnosis. The function of AD biomarkers within a clinical context is depicted in Figure 2.

## 6. Core Biomarkers

**Figure 3.**
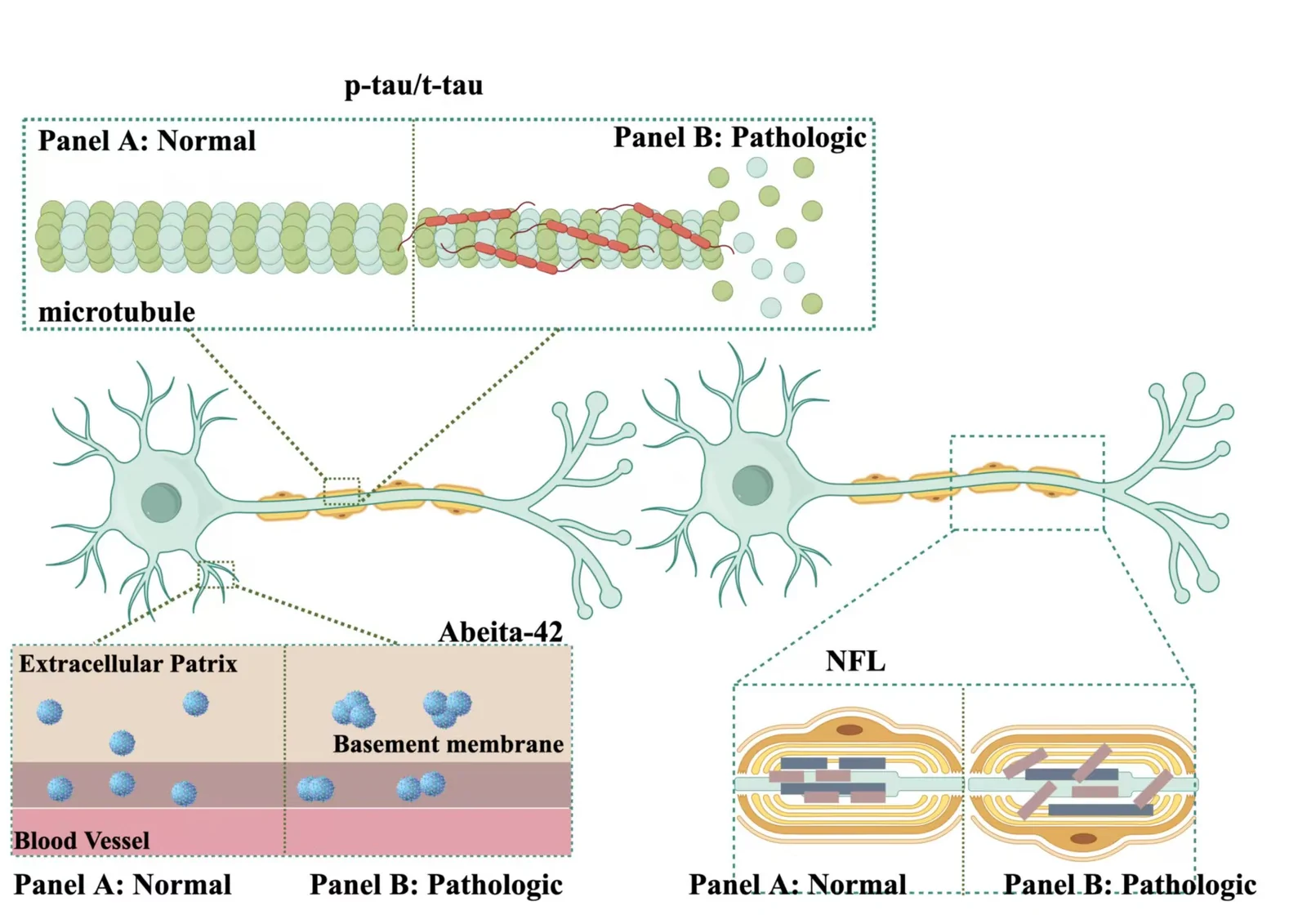
**Depictions of pathophysiology associated with the AD Signature**. (1) Aβ42 is a peptide composed of 42 amino acids, produced through the cleavage of the amyloid precursor protein by beta and gamma secretases. Panel A illustrates the effective removal of smaller amyloid peptides via perivascular basement membranes. Panel B shows the compromised clearance of larger Aβ peptides, which correlates with heightened amyloid accumulation in the brain, leading to the presence of Aβ oligomers in the extracellular matrix and reduced levels of Aβ42 in the CSF. (2) Tau is a protein associated with microtubules that contribute to intracellular NFTs associated with AD. Panel A depicts that tau can be phosphorylated in its functional form, playing a role in the dynamic instability of microtubules. Panel B demonstrates how tau proteins undergo hyperphosphorylation in AD, resulting in the premature detachment of tau from microtubules and disrupting the equilibrium of microtubule assembly and disassembly. (3) Neurofilament light (NfL) is the smallest and most prevalent among the three polypeptides that constitute neurofilament proteins present in large-caliber, myelinated axons. Panel A shows that neurofilaments coexist with microtubules within the axon, enhancing both the axon's diameter and conduction speed. In instances of axonal injury, as shown in Panel B, neurofilaments leak into the extracellular environment and are subsequently removed as cellular debris into the CSF.

The new standard introduced the concept of core biomarkers, categorizing them into Core 1 and Core 2 based on their distinct pathophysiological roles throughout the progression of AD. Core 1 comprised the following biomarkers: CSF Aβ42/40, CSF p-tau181/Aβ42, CSF t-tau/Aβ42, p-tau217 in plasma, the ratio of plasma p-tau217 to n-p-tau217, along with Aβ PET. In contrast, Core 2 included tau PET [26]. According to this standard, the presence of one or more positive indicators in Core 1 fulfills the criteria for the initial biological marker staging of AD (Stage A). A positive finding in the medial temporal lobe on tau PET corresponded to the early biological marker staging of AD (Stage B). Moderate uptake observed in the neocortex on tau PET indicated mid-stage biological marker staging of AD (Stage C), while high uptake signified late biological marker staging of AD (Stage D) [26]. Depictions of pathophysiology associated with the AD Signature were shown in Figure 3.

### 6.1. CSF Biomarkers

In liquid biomarker research for AD, CSF was considered as pivotal research medium due to its close association with the central nervous system (CNS) [27]. CSF accurately reflected the pathophysiological changes within the CNS, exhibiting both high specificity and sensitivity, and has been utilized in the clinical diagnosis of AD [28]. At present, the CSF biomarkers most frequently utilized for AD consist of Aβ, specifically Aβ42, Aβ40, and their hybrids, in addition to total tau (t-tau) and the phosphorylated tau protein, especially p-tau181 [29]. Furthermore, several biomarker panels in CSF have been established to improve diagnostic precision. For AD diagnosis, a reduction in CSF Aβ42 levels alongside a decrease in the Aβ42/Aβ40 ratio were recognized as key indicators within the ATN framework, effectively indicating cerebral amyloidosis.

### 6.1.1. Aβ in CSF

The main pathological indicator of AD is Aβ, which is encoded by the gene for amyloid precursor protein (APP) found on chromosome 21. This protein is generated via the cleavage processes carried out by β-secretase and γ-secretase enzymes [30]. Due to variations in γ-secretase cleavage sites, different forms of Aβ, namely Aβ38, Aβ40, and Aβ42, are generated, with Aβ42 comprising approximately 10% of the total [31]. Aβ42 is particularly prone to oligomerization, leading to its aggregation as the core component of senile plaques, which exhibit significant neurotoxicity and aggregation properties. The primary type of Aβ present in the brains of individuals with Alzheimer's disease is the one typically observed [32]. In a healthy physiological state, there is a balance between the production and breakdown of Aβ. However, excessive production results in deposition, which triggers inflammatory responses in the affected brain tissue. This activation of inflammation-related pathways promotes the release of various inflammatory factors, ultimately leading to neuronal apoptosis and axonal damage, which play a vital role in the advancement of AD [33]. Research demonstrated a reduction in Aβ42 concentrations among patients with AD [34], reflecting Aβ plaque deposition, while an increase in Aβ42 levels is associated with a slowdown in cognitive and clinical decline [35]. Furthermore, Aβ42 can serve as a biomarker for identification of early stages of AD, such as MCI or preclinical AD [36].

As research on AD biomarkers progresses, the ratio of CSF Aβ42 to Aβ40 has become a central focus in contemporary studies. In contrast to simply measuring CSF Aβ42 alone, the Aβ42/Aβ40 ratio provides a more precise depiction of the Aβ pathological features found in the brains of individuals with AD [37, 38]. This made it the most promising Aβ-related metabolic marker. Additionally, the Aβ42/Aβ40 ratio demonstrated strong association with Aβ-PET findings [39-41], indicating its possible use as a surrogate marker for Aβ-PET, especially in scenarios where imaging assessments are not feasible. Clinical studies conducted across multiple centers have shown that the Aβ42/Aβ40 ratio is the sole biomarker that maintains stability in cases of AD [42]. Janelidze et al. [43] utilized single-molecule array technology to analyze 719 large-scale samples, demonstrating that plasma Aβ42 levels and the Aβ42/Aβ40 ratio were considerably reduced in patients with AD compared to healthy subjects and those with MCI. Furthermore, a significant relationship was observed between plasma Aβ levels and CSF Aβ levels in patients with AD, thus validating the diagnostic relevance of plasma Aβ42 and the Aβ42/Aβ40 ratio for this condition. Notably, Aβ42 is susceptible to oligomerization or binding to molecules that hinder the recognition of epitopes by detection antibodies, which can diminish diagnostic efficiency when assessed in isolation. However, using Aβ40 as a standardizing factor for Aβ42 and employing the declining trend of the ratio can more reliably reflect the early pathological changes associated with AD. Zhu et al. [44] conducted a study involving patients with cognitive impairment admitted to the Department of Neurology at Nanjing University of Traditional Chinese Medicine from May 2017 to December 2021, which included 30 patients diagnosed with AD and 19 non-AD patients. An enzyme-linked immunosorbent assay (ELISA) was utilized to investigate the levels of Aβ1-42, total tau protein, and phosphorylated tau protein in the CSF of the patients, while FreeSurfer image analysis software was utilized to calculate the volumes of various subregions of the hippocampus. The findings suggested that, within the AD group, the volume of the right parahippocampal gyrus showed a negative correlation with the concentration of Aβ1-42 in the CSF, while exhibiting a positive correlation with the levels of phosphorylated tau protein.; the volumes of the left hippocampal tail, CA1, parahippocampal gyrus, molecular layer, dentate gyrus, CA3, and CA4 in the AD group were all negatively correlated with the total tau protein concentration in the CSF. In the non-AD group, there was no significant correlation between the volumes of the hippocampal subregions and the concentrations of CSF biomarkers. The significance of Aβ pathological biomarkers present in CSF for the early identification of AD is emphasized by these findings.

### 6.1.2. Tau Protein in CSF

The tau gene, located on chromosome 17 (17q21-q22), encodes a protein known as tau, which is associated with microtubules [45]. The main role of tau protein is to preserve the stability of microtubules. A typical tau protein molecule possesses around 2 to 3 phosphorylation sites; however, in the brains of individuals with AD, tau protein experiences hyperphosphorylation, leading to each molecule having approximately 5 to 17 phosphorylation sites. This hyperphosphorylation leads to microtubule depolymerization and a loss of biological function [46]. In clinical settings, p-tau and t-tau serve as essential biomarkers [47]. The p-tau variant is characterized by its phosphorylation, and a significant increase in its concentration is closely linked to the formation of NFTs in AD [48].

Currently, the primary biomarkers for AD in CSF are the elevated concentrations of Aβ42 and p-tau181 [49], with CSF p-tau181 being the earliest and most extensively studied tau pathology marker, incorporated within the diagnostic guidelines for AD [11]. It holds significant importance in the diagnosis of AD and other associated conditions. Research indicates that CSF p-tau217 outperforms p-tau181 in identifying AD pathology [50-52]. Research indicates that, in contrast to p-tau181, p-tau217 is useful in distinguishing between AD and frontotemporal lobar degeneration (FTLD) syndromes, acts as a marker for Aβ-PET positivity, and exhibits a more robust association with tau-PET signals [53]. CSF T-tau reflected extensive neuronal damage and was served as a non-specific marker for neuronal degeneration, which was applicable across various neurological disorders [54]. Its combined use with Aβ42 and p-tau improves the precision of diagnosing AD. Unlike Aβ, its accumulation in the brain occurs 10 to 20 years prior to the manifestation of cognitive decline symptoms in individuals with AD, the aggregation of Tau primarily begins during the MCI stage and worsens as AD symptoms progress. Consequently, Tau-PET is applicable solely for diagnosing patients with AD, rather than for predictive purposes [5]. Since various neurodegenerative diseases associated with cognitive impairment are linked to elevated t-tau levels, t-tau cannot be regarded as a specific biomarker for AD.

### 6.1.3. CSF biomarkers Panel

Compared to single biomarker testing, combined detection of Aβ imaging and tau imaging was used, specifically, the relationships between CSF t-tau/Aβ42, p-tau/Aβ42, and Aβ42/p-tau181 provided a more precise representation of AD pathology [55-57]. Research indicates that the ratio of CSF Aβ42 to p-tau181 exhibits sensitivity and specificity levels reaching 94% and 90%, respectively, when differentiating AD from cognitively normal (CN) individuals; furthermore, it effectively distinguishes AD dementia from non-AD dementia [58]. This is crucial for forecasting the advancement of MCI to AD. When using Aβ-PET as the gold standard, this ratio demonstrated an accuracy of approximately 90% in differentiating AD from various forms of cognitive impairment in patients with cognitive disorders [55]. Recent studies have found that the concentrations of CSF Aβ42 and the ratio of Aβ42 to Aβ40 show an inverse relationship with the burden of cerebral amyloid β plaques. In contrast, the levels of t-tau and p-tau are related to the degree of neurodegeneration and the occurrence of NFTs, respectively [59]. The results of this research offer a more thorough and strong foundation for accurately diagnosing and evaluating the condition of AD. Key fluid biomarkers associated with AD consist of Aβ pathology and tau pathology unique to the disease. Moreover, CSF biomarkers related to synaptic function, along with those indicative of calcium imbalance and axonal damage, are crucial in the assessment and diagnosis of AD. However, the acquisition of CSF samples presents certain limitations. This process requires an invasive procedure known as lumbar puncture [60], which not only inflicts physical trauma on patients but also carries risks such as infection, bleeding, and headache. Furthermore, the expense associated with this examination is quite substantial, which adds to the mental burden and financial pressure on patients, resulting in generally low acceptance in clinical practice.

### 6.2. Biomarkers in Blood

Both CSF and plasma can indicate the pathological states present in a patient's brain. However, obtaining blood samples is relatively simpler, as they can be collected multiple times, which enhances their acceptability among patients. Consequently, measurements of plasma AD biomarkers are easier to obtain compared to CSF tests and PET scans. Blood biomarkers can be used to detect the likelihood of developing AD, track the transition from MCI to AD, and indicate the swift progression of AD that has been clinically diagnosed [61]. They are appropriate for extensive screening and ongoing observation of AD, playing a crucial role in early detection, diagnosis, and subsequent monitoring of this condition [62]. With the development of highly sensitive and precise detection methods, research on peripheral blood biomarkers in AD has rapidly advanced, allowing for the detection of changes in peripheral blood biomarker levels even in the preclinical stages of AD. The investigation of novel peripheral blood biomarkers offers new insights and broader prospects for risk prediction, diagnosis, and differential diagnosis of AD.

### 6.2.1. Aβ in Blood

In recent years, the research on Aβin blood has increased significantly, and its potential as a diagnostic tool for AD has progressively been acknowledged [63]. Compared to CSF, blood tests exhibited substantial advantages. The Aβ proteins in blood primarily consist of three types: Aβ38, Aβ40, and Aβ42. Currently, the majority of researchers agree that the ratio of plasma Aβ42 to Aβ40 is more effective for diagnosing AD than assessing Aβ40 or Aβ42 separately [64]. The post-mortem studies have shown a strong correlation between the plasma Aβ42/40 ratio and the pathological alterations of Aβ in the brain [65]. A 2019 study found that the plasma Aβ42/40 ratio exhibited high precision in identifying abnormal Aβ deposition in the brain [66]. Palmqvist et al. [67] demonstrated that the plasma Aβ42/40 ratio effectively reflected the irregularities observed in the CSF Aβ42/40 ratio among CN individuals, patients with MCI, and those suffering from AD dementia (AUC=0.81-0.87). This finding supported the reliability of the plasma Aβ42/Aβ40 ratio in diagnosing AD [67]. Furthermore, research indicates that a lower Aβ42/Aβ40 ratio correlates with diminished cognitive performance in individuals [68]. The plasma Aβ levels can be used to distinguish AD-type dementia from MCI patients [49]. However, in individuals with well-defined Aβ pathology, the reduction in CSF Aβ42/40 ranged from 40% to 60%, while the decrease in plasma Aβ42/40 was only between 10% and 20% [69]. This suggested that when analyzing plasma Aβ42/Aβ40 levels, they may be more susceptible to factors such as sampling environment, storage conditions, and the duration of sample delivery, all of which can potentially alter plasma Aβ42/Aβ40 levels. The differences observed in the research outcomes may also be linked to the existence of the blood-brain barrier (BBB), resulting in reduced levels of blood biomarkers.

If the sensitivity of the detection method is insufficient, accurately measuring trace amounts of Aβ in peripheral blood becomes challenging. Additionally, the high abundance of proteins in the blood may interfere with the analysis process, thus affecting the detection accuracy [70]. It was indicated that Aβ and tau proteins present in serum neuronally-derived exosomes (NDE) can penetrate the BBB into peripheral blood without interference from other physiological factors. Neuron-derived exosome can be analysed for minimally invasive capture of CNS pathology through blood. This characteristic allows for a more precise representation of the neuropathological alterations in the brain tissue of patients with AD [71]. Research has also indicated that plasma levels of oligomeric Aβ may serve as promising blood biomarkers for the early identification of AD. These markers could not only provide accurate predictions for PET diagnostic outcomes (AUC = 0.855) [72], but they could also exhibit a strong correlation with cognitive abilities, enabling the differentiation between patients with AD and CN individuals [73]. Schindler et al. [66] reported that a diminished plasma Aβ42/Aβ40 ratio was observed in CN individuals, exhibiting the likelihood of detecting Aβ pathology via PET increased 15-fold over a 4-year period. A study comparing the sequence of changes in plasma and CSF biomarkers in AD patients with PET findings revealed that the plasma Aβ42/Aβ40 ratio detected Aβ abnormal deposition earlier than CSF and PET, demonstrating its greater value for early clinical diagnosis [74]. However, the standard critical value for Aβ42/Aβ40 ratio detection has yet to be established, and enhancing its accuracy for early AD diagnosis poses more challenges. A research investigation revealed that establishing the critical threshold for the plasma Aβ42/Aβ40 ratio at 0.089 results in a diagnostic AUC of 0.79. This threshold is associated with a sensitivity of 85%, a specificity of 63%, a positive predictive value of 81%, and a negative predictive value of 70%. Following the inclusion of age and apolipoprotein E (APOE) genotype in the statistical model, the overall accuracy increased to 83% [75]. Chatterjee et al. [76] discovered that several measurement factors affect the Aβ42/Aβ40 ratio. Additionally, the presence of p-tau181 and GFAP can improve the precision in predicting the likelihood of progression to MCI and AD in healthy individuals. This suggests that a multi-biomarker combination model will improve diagnostic performance.

### 6.2.2. Tau Protein in Blood

p-Tau and t-tau are internationally recognized as crucial biomarkers for the diagnosis of AD, with CSF levels of t-tau and p-tau included in the international diagnostic guidelines for the condition [77]. The p-tau protein contains over 40 phosphorylation sites [53]. Based on the differences in these phosphorylation sites, p-tau is primarily classified into three subtypes: p-tau217, p-tau181, and p-tau231 [78]. Extensive research has been conducted on the p-tau181 subtype, demonstrating a significant correlation between elevated levels of p-tau181 in the blood and AD pathology [5]. Studies indicated that it can accurately predict pathological changes in tau protein and Aβ, as well as effectively distinguished AD from other neurodegenerative diseases, making it valuable for identification of AD across various clinical stages. Notably, p-tau181 may serve as an indicator of the likelihood of developing future AD, as it can anticipate cognitive deterioration and hippocampal shrinkage within a one-year period [79]. Research conducted by Janelidze et al. [80] revealed that p-tau181 plasma concentrations start to increase in the preclinical phase of AD and persist in rising throughout the stages of MCI and dementia. Additionally, these plasma levels of p-tau181 exhibit a significant ability to distinguish between AD and non-AD dementias.

In recent years, researchers have identified that p-tau217 demonstrates higher specificity in comparison to p-tau181. The concentration of p-tau217 accurately reflected the underlying tau pathology, holding significant implications for the clinical diagnosis of AD [81]. Research conducted on three selected cohorts comprising 1,402 participants revealed that the diagnostic accuracy of plasma p-tau217 for clinical AD was notably superior to that of plasma p-tau181, plasma NfL, and MRI assessments. Plasma levels of p-tau217 significantly correlated with tau tangles presented in the brain and are capable of effectively distinguishing abnormal tau PET outcomes from normal ones. Furthermore, plasma p-tau217 demonstrated significant precision in differentiating AD from other neurodegenerative disorders, with its performance comparable to key indicators based on CSF or PET, and was significantly superior to established plasma and MRI biomarkers [82]. It was suggested that plasma p-tau217 may reduce the necessity for invasive lumbar punctures while maintaining the precision of AD identification [83]. Additionally, a study based on a Swedish sample found that p-tau217 can predict with nearly 100% accuracy on whether a subject has AD [84]. Devanarayan et al. [85] investigate whether the ratio of phosphorylated plasma Tau217 (pTau217R) can serve as a predictor for tau accumulation across various brain regions, assessed through the PET standardized uptake value ratio (SUVR), which is crucial for AD staging. The measurement of plasma pTau217R was conducted using immuno-precipitation-mass spectrometry [85]. Findings from the predictive models for tau PET SUVR indicated that the pTau217R model surpassed those based on clinical data, MRI scans, and additional blood biomarkers. This model consistently forecasted tau levels that were above the tau positivity threshold and other elevated benchmarks. Utilizing pTau217R for screening could lessen the requirement for tau PET scans by 65%, achieving a sensitivity rate of 95% [85]. Additionally, the pTau217R model is beneficial for disease staging and monitoring in the early stages of AD. The plasma p-tau217 also demonstrates a high level of accuracy in differentiating AD from non-AD dementia.

It was indicated that CSF p-tau231 may serve as an early marker for emerging pathological changes associated with AD [67]. Subsequently, plasma p-tau231 has been confirmed as a potential biomarker [86]. Both plasma p-tau231 and p-tau217 function as preclinical markers for AD. Among these, plasma p-tau231 may be particularly suitable for evaluation of middle-aged individuals who have experienced alterations in soluble Aβ, even if the level of Aβ pathology detectable by PET has not yet surpassed the threshold [87]. Notably, p-tau231 shows a considerable ability to differentiate AD from non-AD dementia, reaching an AUC of 0.93. Additionally, it effectively distinguishes AD from MCI cases, with an AUC of 0.89 [88]. The reduction of the Aβ42/Aβ40 ratio, coupled with the elevation of p-tau217 and p-tau181, was significantly associated with the diagnostic process for AD. These biomarkers exhibited high potential for clinical application, and it is expected that improvements in detection technologies will further enhance their efficacy in the future. Recent discoveries regarding p-tau205, which differs from traditional p-tau proteins, indicated a close association with NFTs rather than Aβ. This indicates that p-tau205 has a stronger association with the pathological tau burden present in the brain.The Braak staging model is employed in pathology to describe the distribution and progression of NFTs in the brain [89]. This staging was determined through immunostaining with the AT8 antibody, which primarily binds to phosphorylated tau at threonine 205 and serine 202 [90]. Consequently, p-tau205 and p-tau202 might offer a more precise indication of the condition of NFTs in the brains of AD patients when contrasted with other phosphorylated tau epitopes.

### 6.2.3. Blood biomarkers Panel

In a longitudinal cohort study involving 145 elderly Brazilians, Santos et al. [91] evaluated the diagnostic efficacy of plasma biomarkers in relation to clinical diagnoses and the positivity of CSF biomarkers. The SIMOA HD-X platform was utilized to measure plasma concentrations of Tau, Aβ40, Aβ42, NfL, GFAP, pTau231, p-tau181, and p-tau217. Findings revealed that plasma p-tau217 demonstrated exceptional capability in identifying CSF biomarker status within the studied cohort, both independently (ROC AUC = 0.94) and in comparison, to Aβ42 (ROC AUC = 0.98) [91]. Thijssen et al. [92] investigated a set of blood-based biomarkers, namely Aβ1-42/1-40, p-tau181, NfL, and GFAP, which serve to differentiate between AD, frontotemporal dementia (FTD), and dementia with Lewy bodies (DLB). Their findings revealed that the Simoa panel, which effectively distinguished AD from FTD, included NfL and p-tau181 (AUC = 0.94) or featured NfL, GFAP, and p-tau181 (AUC = 0.90). In the context of separating AD from DLB, the panel consisted of NfL, p-tau181, and GFAP (AUC = 0.88), with p-tau181 alone also providing a differentiation (AUC = 0.81) [92]. These findings indicated that a combination of plasma p-tau181, NfL, and GFAP, but not Aβ1-42/1-40, may be useful for discrimination between AD, FTD, and DLB.

Single biomarkers may be influenced by physiological fluctuations or non-disease factors. In contrast, a panel that combines multiple biomarkers reduces the rates of false positives and false negatives. Furthermore, it facilitates the batch processing of samples, rendering it suitable for biomarker screening in epidemiological surveys or clinical trials of drugs, thus possessing irreplaceable clinical value in early disease intervention. With advancements in detection technologies, the application scenarios for such panels will continue to expand.

## 7. Other Body Fluid Biomarkers.

### 7.1. Saliva

Numerous substances are absorbed from capillaries into saliva, making saliva a significant "reflection" of the human physiological state [93]. The secretion process of the salivary glands is regulated by the autonomic nervous system, and proteins associated with the CNS are also present in saliva, with their levels strongly correlated with age [94]. The phenomenon of reduced salivary flow in the elderly is relatively common and may be attributed to the effects of medication or a decline in salivary gland function due to aging. Although research has identified key biomarkers such as Aβ and p-tau in saliva, these indicators cannot currently be considered reliable bases for diagnosing AD [95]. While Aβ40 and t-tau levels in saliva do not have a direct connection to clinical AD, there may be a potential relationship between elevated Aβ42 and p-tau, as well as decreased lactoferrin. Notably, lactoferrin, an antimicrobial peptide, has been demonstrated with a strong correlation with biomarkers in the CSF of AD and could potentially be utilized for detecting preclinical stages of AD [96]. Elderly patients, particularly those with dementia, often struggle to provide adequate saliva samples. As a result, salivary biomarkers are more suitable for the early stages of the disease compared to populations with significantly impaired cognitive abilities. Saliva serves as a non-invasive source of biomarkers, offering greater accessibility and circumventing the ethical concerns associated with blood and CSF collection. Additionally, saliva contains a wealth of practical disease biomarkers, making it promising for the preclinical stages of diseases, and it presents significant advantages for the future diagnosis of conditions such as AD. However, further research is necessary to establish its reliability in detecting disease pathology and monitoring disease progression.

### 7.2. Urine

The composition of urine reflects the cellular components, biochemical compounds, and proteins filtered through the glomeruli and excreted by the renal tubules from plasma, serving as an indicator of an individual's metabolic and pathophysiological status [97]. Numerous research efforts indicated a significant correlation between neurotrophic factor (NTP) and neuronal growth and degeneration. Furthermore, expression levels of NTP may be increased in the brain tissue, CSF, and even in the urine of patients with AD [98]. Specifically, multicenter studies have demonstrated that the neuronal thread protein associated with AD (AD7c-NTP) present in urine exhibited considerable sensitivity and specificity for diagnose of AD [99]. The research conducted by Zhang et al. [100] suggested that urine levels of AD7c-NTP were elevated in patients with AD and MCI who exhibited cerebral deposition of Aβ. The urinary biomarker AD7c-NTP was demonstrated to have strong specificity for predicting Aβ accumulation in individuals with cognitive deficits. Furthermore, its concentrations was shown with a positive correlation with the progression of AD [101]. However, the specific cut-off values for its diagnostic use would require further in-depth investigation. Currently, the AD7c-NTP testing kit has been received approval from the National Medical Products Administration for early clinical detection of AD and for identification of individuals in high-risk groups. Additionally, the elevated levels of formaldehyde (FA) and formic acid in urine were also considered as potential biomarkers for AD. FA levels were associated with various cognitive deficits, including AD. A meta-analysis performed by Chen et al. [102] revealed that FA levels in patients with AD were significantly increased, potentially linking them to the disease's pathological processes and suggesting their possible utility as supportive diagnostic biomarkers in clinical settings.

Wang et al. [103] included a total of fifty-seven individuals diagnosed with AD, 43 patients with MCI, and 62 CN participants. They performed an analysis using liquid chromatography-tandem mass spectrometry (LC-MS/MS) on urine samples. The investigation identified 33 proteins that were significantly different between the AD and CN groups. The diagnostic panel for AD comprised DDC, CTSC, EHD4, GSTA3, SLC44A4, GNS, GSTA1, ANXA4, PLD3, CTSH, HP, RPS3, CPVL, age, and the APOEε4 allele, which yielded an AUC of 0.9989 in the training cohort and 0.8824 in the validation cohort [103]. Following comprehensive traditional statistical analyses and bioinformatics investigations, a novel diagnostic panel for AD has been identified, comprising 30 metabolites, age, and the presence of the APOEε4 allele. This urine-based diagnostic panel offers clinicians a convenient, non-invasive, and valuable method for differentiating AD from CN individuals. Among the metabolites identified, Atropine, M6P, and PLP have been recognized as evidence-based hub metabolites in AD. However, the roles of S-Methyl-L-cysteine-S-oxide, Spiculisporic Acid, N-Acetyl-L-methionine, 13,14-dihydro-15-keto-tetranor Prostaglandin D2, and 17(S)-HpDHA in AD require further investigation [104]. Urine, as an ideal source of AD biomarkers, offers advantages such as ease of collection and non-invasiveness.

### 7.3. Tears

Tears are an exocrine secretion produced by the lacrimal glands [105]. Research indicates that patients with neurodegenerative diseases, including AD, often experience dysfunction in tear secretion [106]. Diminished secretion of tears may act as an initial indicator of cognitive decline. In a certain cross-sectional investigation, 35 participants were involved: 11 diagnosed with prodromal AD classified as having MCI, 10 with mild to moderate AD, and 14 HCs [107]. After obtaining tear samples, the concentration of Aβ1-42 was evaluated through the ELISA method. Findings reveal that tears containing Aβ1-42 can effectively identify both MCI and AD individuals, with a specificity of 93% and a sensitivity of 81% (AUC = 0.91) [107]. Furthermore, Aβ1-42 levels in tears were significantly reduced in both MCI and AD participants compared to the healthy controls. Thus, measuring Aβ1-42 levels in tear samples appears to be an unobtrusive and economical strategy for the early identification and diagnosis of AD [107]. Research carried out by Liang et al. [108] demonstrated a relationship between reduced tear production and cognitive decline, indicating that tears may serve as a possible biomarker for the diagnosis of cognitive disorders. Kenny et al.'s [109] involved a total of 32 donors, including 9 individuals diagnosed with AD, 8 with MCI, and 15 age-matched controls. Researchers evaluated the protein content in tears utilizing LC-MS, while the profiling of microRNA content was performed using a genome-wide, high-throughput PCR platform [109]. Their findings highlighted elongation initiation factor 4E (eIF4E) as a distinct protein that was exclusively found in samples from AD patients [109]. In addition, a higher overall abundance of microRNAs was observed in the tear samples of those with AD. Notably, microRNA-200b-5p emerged as a promising biomarker for AD, with significantly elevated levels detected in the tear fluid of AD patients compared to the control group [109]. This research indicates that tear fluid could represent a novel reservoir of biomarkers for AD. The identification and verification of these biomarkers in tears could facilitate the development of a non-invasive and cost-effective diagnostic test for AD. Nonetheless, the limited volume of tear fluid and the constraints of current detection technologies necessitate further research to validate the diagnostic value of these markers.

## 8. AD Novel Biomarkers

### 8.1. Neurofilament Light Chain (NfL)

As a fundamental protein constituent of the axonal cytoskeleton, NfL has been receiving significant recognition as a biomarker due to its sensitivity in indication of axonal injury across a spectrum of neurological disorders. The neurofilament (NF) family comprises four distinct subunits: NfL, medium chain neurofilament (NFM), heavy chain neurofilament (NFH), and α-internexin, which collectively form an intermediate filament network within the cytoplasm of neurons. Among these, NfL, being the smallest and most abundant subunit in the NF family, is highly expressed in myelinated axons and plays a crucial role in maintaining axonal integrity and stable neural conduction [110]. Furthermore, NfL serves as a potential biomarker for the early detection of dementia [111].

Research indicates that levels of NfL in the CSF of people with AD are considerably higher than those observed in cognitively healthy individuals [112]. Recent developments in detection technologies have made it possible to measure NfL levels in blood, thereby enabling more thorough investigations into the connection between plasma NfL and AD. NfL has been suggested as a possible blood biomarker for neurodegeneration related to dementia, since it directly indicates the severity of neuronal degenerative injury [61]. Notably, NfL exhibits a strong correlation between CSF and blood, with correlation coefficients (r values) varying from 0.70 to 0.97, establishing it as the first neuro-specific biomarker proven to have clinical value [5]. However, due to elevated NfL levels in various neurodegenerative diseases, its specificity is relatively low, which limits its use as an independent biomarker for diagnosing AD. Nevertheless, given that NfL levels correlate strongly with disease advancement and the speed of brain shrinkage in the majority of neurodegenerative disorders, it remains an effective measure of neurodegenerative intensity in AD.

Notably, it was demonstrated that individuals carrying mutations in the PSEN1 or APP genes exhibited significantly elevated plasma concentrations of NfL compared to non-carriers [113]. Furthermore, individuals with these mutations began to show increased plasma NfL levels up to 15 years before the onset of AD symptoms, with concentrations progressively rising over time [114]. This finding suggested that plasma NfL may have significant potential for screening AD in asymptomatic high-risk populations. Furthermore, the research indicated a robust correlation between plasma NfL levels, CSF NfL levels, cognitive ability, and the degree of brain atrophy. In individuals without dementia who possessed a genetic predisposition to AD, a significant relationship is observed among cognitive function, plasma NfL levels, and tau pathology [115]. The concentration of plasma NfL can be employed to identify patients with AD as early as a decade or more before the onset of symptoms, this finding aligns with the accumulation of brain PET imaging and CSF biomarkers indicative of brain injury [116].

### 8.2. Neurogranin

Neurogranin (Ng) is a unique protein primarily found in the dendritic structures of excitatory neurons, especially in the cortex and hippocampus regions [117]. It significantly contributes to the management of long-term potentiation (LTP) and is closely associated with synaptic loss in individuals affected by AD. Synaptic impairment is recognized as a significant pathological mechanism in AD. During the initial phases of AD progression, there is a reduction in synapses accompanied by neuronal damage. Ng, a protein that affects synaptic plasticity, is essential in this mechanism. Studies show that the levels of Ng found in the CSF of patients with AD are markedly elevated compared to those in healthy people. [118]. This increase in Ng levels may signify synaptic dysfunction and neuronal degeneration. In the CSF of individuals with AD, Ng concentrations are significantly increased, which corresponds to reductions in cognitive functions and brain shrinkage. Agnello et al. [119] performed a retrospective observational study that included 29 patients diagnosed with AD and 59 patients without AD, all of whom underwent lumbar puncture for CSF analysis. Neurogranin and α-synuclein levels in the CSF were assessed through a highly sensitive ELISA method. The research indicated that patients diagnosed with AD exhibited notably higher concentrations of neurogranin and α-synuclein in their CSF compared to individuals without the disease. Additionally, a correlation was found between neurogranin levels and both t-tau and p-tau, alongside a relationship with cognitive decline in AD patients [119]. The analysis of the ROC curve indicated that neurogranin has a high diagnostic accuracy in the identification of AD, featuring a cutoff value of 306 pg/mL, an AUC of 0.872, and sensitivity and specificity rates of 84.2% and 78%, respectively [119].

In the research carried out by Piccoli et al. [120] the levels of CSF Ng and α-Syn were assessed in individuals diagnosed with AD (n = 69), those without Alzheimer's (non-AD) (n = 50), and patients with non-degenerative disorders (ND) (n = 98). The results indicated that the concentrations of CSF Ng and α-Syn were significantly elevated in AD patients compared to both n-AD and n-ND groups [120]. Furthermore, the ratios of Aβ42/Ng and Aβ42/α-Syn exhibited statistically significant differences among the groups, effectively distinguishing AD patients from those with n-AD, outperforming Ng or α-Syn alone in this regard. Additionally, regression analyses revealed a correlation between higher Ng concentrations and a Mini-Mental State Examination (MMSE) score of less than 24, pathological Aβ42/40 ratios, p-Tau, t-Tau, and the *APOEε4* genotype [120]. The Aβ42/Ng ratio was also associated with an MMSE score of less than 24, an FDG-PET pattern associated with AD, the *APOEε4* genotype, levels of pathological Aβ42, along with p-Tau and t-Tau. Notably, *APOEε4* carriers exhibited higher Ng concentrations compared to non-carriers [120]. Compared to various other neurodegenerative diseases, Ng shows a higher specificity in diagnosing AD, thus serving as a valuable biological marker for the accurate diagnosis of AD [121].

### 8.3. BACE1

BACE1 serves as the primary enzyme responsible for the production of Aβ peptides, playing a pivotal role in this process. As a type I transmembrane aspartyl protein kinase, BACE1 is implicated in the myelination of peripheral nerve cells and is extensively distributed within the Golgi apparatus of various tissue types, exhibiting the highest activity in nerve cells [122]. All monomeric variants of Aβ, including Aβ42, require the presence of BACE1 [123]. It is crucial in the initial cleavage step of the APP protein; the subsequent continuous action of γ-secretase, along with other processes, leads to the generation of Aβ-40 and Aβ-42 peptides. The buildup of these peptides leads to the development of senile plaques, a hallmark of AD, which ultimately causes neuronal death and a deterioration in cognitive abilities [124].

According to Shen et al. [125], the BACE1 protein and its activity markers show promise as potential blood biomarkers for a range of diseases, including AD. This protein can be detected in plasma, where its concentration was significantly increased in individuals with MCI. Furthermore, this metric has the potential to forecast the transition from MCI to AD. BACE1 is the primary catalyst for the production of Aβ and is predominantly found in the brain, particularly within neurons, oligodendrocytes, and astrocytes [126]. Both the expression levels and enzymatic activity of BACE1 were markedly elevated in the brains and bodily fluids of AD patients [127]. To determine if lncRNA BACE1-AS could act as a blood-based biomarker for AD, Fotuhi et al. [128] analyzed the levels of this molecule in plasma and exosomes derived from plasma in both AD patients and healthy controls. The findings validated the existence of BACE1-AS in plasma samples from both groups. Importantly, substantial differences were observed between the AD subgroups and the control groups in the overall plasma samples. Specifically, the level of BACE1-AS was observed to be reduced in the pre-AD subgroup, whereas it was increased in those with full AD when compared to healthy control subjects [128]. Additionally, the analysis of the ROC curve suggested that lncRNA BACE1-AS could reliably differentiate pre-AD individuals from healthy controls (with sensitivity at 75% and specificity at 100%), distinguish full-AD patients from healthy controls (showing 68% sensitivity and 100% specificity), as well as differentiate between pre-AD and full-AD subgroups (possessing 78% sensitivity and 100% specificity). This highlights its potential utility as a biomarker in tracking the progression of AD [128]. To conclude, the plasma concentrations of BACE1-AS might serve as a valuable blood-based biomarker for AD.

### 8.4. Synaptosome-associated protein 25 (SNAP-25)

The protein synaptosome associated protein 25 (SNAP-25) is a crucial presynaptic component, it significantly influences the fusion of synaptic vesicles triggered by calcium and the subsequent release of neurotransmitters into the synaptic cleft [129]. In the early stages of many neurodegenerative disorders, the loss of synapses is a common characteristic. Synaptic dysfunction was regarded as a key mechanism underlying cognitive impairment, particularly in AD [130]. During both the initial phases and the progression of AD, a persistent decrease in synaptic density and connectivity has been observed across various brain regions [131]. This synaptic loss preceded neuronal degeneration and resulted in alterations to presynaptic terminal proteins such as SNAP-25, which have been found to be elevated in the CSF of patients with AD, while no significant changes were noted in other neurodegenerative conditions.

Research conducted by Halbgebauer et al. [132] investigated the levels of SNAP-25 across various neurodegenerative disorders using the ELISA immunoassay technique, revealing increased SNAP-25 concentrations in the CSF during the initial stages of AD. The study developed a highly sensitive ELISA to assess SNAP-25 levels in CSF and examined SNAP-25 concentrations in groups of patients suffering from different neurodegenerative conditions. This ground-breaking immunoassay method supports the conclusion that cerebrospinal fluid SNAP-25 functions as an important biomarker for AD in both its initial and later stages. Additionally, research conducted by Agliardi et al. [133] demonstrated that exosomes from neurons carry SNAP-25 in serum, suggesting its possible role as a biomarker for AD. Zhang et al. [134] stratified 139 individuals from the Alzheimer's Disease Neuroimaging Initiative (ADNI) database into four groups: CN, stable MCI, progressive MCI, and dementia due to AD. The results indicate that CSF concentrations of SNAP-25, along with the SNAP-25/Aβ42 ratio, were markedly higher in individuals with progressive MCI and AD in comparison to those who are cognitively healthy. Moreover, individuals with normal cognitive function who transitioned to MCI or AD during the follow-up period showed a greater SNAP-25/Aβ42 ratio when compared to those who did not progress. Furthermore, both CSF SNAP-25 levels and the SNAP-25/Aβ42 ratio emerged as significant predictors for the shift from MCI to AD. In patients diagnosed with AD, SNAP-25 levels are associated with the degree of cognitive deficits, as evidenced by MMSE scores. This indicates that assessing serum SNAP-25 concentrations may prove to be a useful metric for tracking cognitive decline in AD patients.

Currently, analyzing t-tau and p-tau levels in CSF reveals indications of neurodegeneration and tau-associated pathological changes, whereas the evaluation of Aβ indicates the presence of amyloid pathology. These evaluations have been effectively integrated into existing diagnostic standards and were utilized in clinical settings. However, these biomarkers could not provide insights into synaptic degeneration, a condition that frequently affects individuals with early attention deficit disorder and MCI. Consequently, SNAP-25 found in CSF represented a promising synaptic biomarker, offering significant potential for the early identification of AD. However, in a discovery cohort study, Saloner et al. [135] found that the C-allele of the SNAP-25 rs1051312 (T > C) variant is associated with increased basal expression of SNAP-25. Carriers of the C-allele demonstrated enhanced verbal memory in healthy women, while this effect was not observed in men. Additionally, female C-carriers showed the lowest levels of amyloid-beta PET positivity. These findings suggest that the SNAP-25 gene could play a role in promoting resistance to AD [135]. Consequently, the investigation of SNAP-25 as a potential biomarker for AD is still in the early stages. Existing studies are limited to scientific research and have not yet been widely promoted in clinical settings. Future research is required to explore and support this area, which holds tremendous potential.

## 8.5. Inflammation-related factors

### 8.5.1. Cytokines

In recent years, it has been elucidated that the pathological mechanisms of AD are intricately linked to immune responses within the brain [136], sharing common pathophysiological bases such as inflammatory responses. Chronic neuroinflammation was regarded as a significant factor in the progression of AD. Cytokines such as interleukin (IL), interferon-gamma, and tumor necrosis factor-alpha (TNF-α) are essential in promoting the immune response within the brains of those affected by AD. For instance, individuals suffering from AD show a significant rise in inflammatory cytokines present in their blood, such as IL-6, IL-8, IL-23, and IL-33. Importantly, IL-8 functions as an inflammatory cytokine, exhibiting characteristics that are both anti-inflammatory and pro-inflammatory. Furthermore, the IL-33/ST2 signaling pathway may hold significant implications for the clinical diagnosis and treatment of AD [137]. One of the key inflammatory agents involved in the nervous system's inflammatory response is TNF-α, which significantly contributes to the inflammatory processes linked to the advancement of AD. Furthermore, the buildup of irregular protein clusters has the potential to activate microglial cells, which in turn results in the release of pro-inflammatory cytokines like TNF-α and IL-1β [138]. This process is accompanied by various pathophysiological alterations, including mitochondrial damage, lysosomal dysfunction, and oxidative stress [139]. It has been demonstrated that mitochondria were crucial in the development of neurodegenerative diseases driven by inflammation [140]. Moreover, neuroinflammation was considered as a link between amyloid accumulation, Tau pathology, and the neurodegeneration process [141].

### 8.5.2. Glial fibrillary acidic protein (GFAP)

GFAP, a protein belonging to the intermediate filament family and found in the cytoskeletal structure of astrocytes, is generated and emitted in large quantities into the bloodstream by reactive astrocytes under conditions of neuroinflammation. Notably, GFAP exhibits a high degree of brain specificity, with significantly elevated levels observed in the brains of individuals with AD, correlating with the rate of cognitive decline [142]. Research indicates that plasma GFAP can predict the risk of dementia up to 15 years in advance [143]. Furthermore, GFAP is expressed at higher levels in regions surrounding Aβ deposition, making it a more accurate predictor of cerebral Aβ accumulation compared to NfL and the Aβ42/Aβ40 ratio [144]. Consequently, GFAP may serve as a sensitive plasma biomarker reflecting the pathological accumulation of Aβ. Nonetheless, it is crucial to acknowledge that GFAP exhibits low specificity since its levels can also rise in several other circumstances, such as spinal cord disorders and central nervous system injuries. Chatterjee et al. [49] measured GFAP using the Single Molecule Array (Simoa) platform and performed a cross-sectional analysis throughout the continuum of AD. This research compares cognitively unimpaired participants who were negative for Aβ-PET with those diagnosed with mild cognitive impairment against Aβ-PET positive individuals from the Australian Imaging, Biomarker & Lifestyle Flagship Study of Ageing (AIBL) cohort. Findings revealed that, over time, altered levels of plasma GFAP and NfL were observed in AD in contrast to cognitively unimpaired people. Furthermore, higher levels of GFAP were linked to future cognitive decline, a reduced plasma Aβ1-42/Aβ1-40 ratio, and an increase in Aβ-PET load over a period. The research conducted by Wagemann et al [145] explores the longitudinal changes in biomarkers linked to neuroinflammation, synaptic dysfunction, and neurodegeneration among individuals with Dominantly Inherited AD who are receiving anti-amyloid treatment. Between 2012 and 2019, the Dominantly Inherited Alzheimer Network Trial Unit (DIAN-TU-001) carried out a randomized, double-blind, placebo-controlled clinical trial to assess the effectiveness of gantenerumab and solanezumab in patients with Dominantly Inherited AD. Findings revealed that gantenerumab resulted in a significant reduction in CSF neurogranin levels by year 4 and lowered plasma GFAP levels in years 1, 2, and 4 when compared to the placebo group. Furthermore, solanezumab was shown to significantly elevate CSF NfL levels at year 4.

In the phase III clinical trial of lecanemab (Clarity AD), plasma p-tau181, Aβ42/40 ratio, and GFAP were used as key secondary endpoints to assess the efficacy of lecanemab. The phase II clinical trial of donanemab (Trailblazer ALZ) employed plasma Aβ42/40 ratio, p-tau217, and GFAP as reference indicators for efficacy monitoring. Similar to CSF, non-AD core biomarkers in blood, such as NfL and GFAP, can differentiate between cognitively impaired patients and cognitively normal individuals; however, these biomarkers lack AD specificity. Among them, Roche's blood GFAP detection kit (Elecsys® GFAP) has been clinically applied. However, as not yet approved by the FDA, it was classified as a research biomarker.

### 8.5.3. YKL-40

YKL-40, also known as chitinase-3-like protein 1 (CHI3L1), serves as an inflammatory marker that is released into the extracellular space in response to inflammatory cytokines, including TNF-α, INF-γ, IL-1β, and IL-6. Research has shown that levels of YKL-40 are elevated in the brains of individuals with AD, especially in the areas surrounding Aβ plaques, as well as in the CSF. Additionally, increased concentrations of YKL-40 have been detected in the serum of individuals suffering from vascular dementia or a combination of dementia types. Similarly, patients diagnosed with MCI exhibit notably higher serum levels of YKL-40, with these values escalating alongside disease advancement and transition to AD. YKL-40, produced by microglia and astrocytes within the brain, plays a role in clearing toxic accumulations of Aβ protein. A meta-analysis has shown marked variations in YKL-40 concentrations in the cerebrospinal fluid and plasma of AD patients when compared to those who are healthy; however, no significant differences were observed in serum levels [146]. The presence of YKL-40 in CSF serves as a marker of healthy aging, and its detection, when combined with core AD biomarkers, can substantially enhance diagnostic accuracy for AD [147]. This finding suggesed that plasma YKL-40 may act as a potential biomarker for AD, highlighting the significance of inflammatory states and glial activation in the disease, and offering new avenues for future therapeutic strategies. Nonetheless, YKL-40 is not exclusively associated with AD dementia; integrating it with additional biomarkers like Aβ1-42, t-tau, and p-tau may improve its diagnostic efficacy.

### 8.5.4. CX3CL1

Fractalkine (FKN), also known as CX3 chemokine ligand 1 (CX3CL1), is primarily a transmembrane protein found in neurons [148, 149]. In the central nervous system (CNS), FKN is predominantly found in the hippocampus [150], where it engages with the receptor CX3CR1, which can be expressed by both neurons and microglia [151]. CX3CL1 is essential for diminishing the activation of microglia and suppressing the expression of pro-inflammatory cytokines and genes, including IL-1β, IL-6, and TNF-α [152]. This action helps maintain the hippocampal microenvironment in a quiescent and anti-inflammatory state [153-155]. Nonetheless, specific triggers and circumstances, such as the buildup of extracellular Aβ and the activation of inflammatory pathways, have the potential to change CX3CL1/CX3CR1 signaling [156]. Moreover, it has been proposed that dysfunctional FKN signaling may predispose microglia to priming following activation. The CX3CL1/CX3CR1 pathway is vital for keeping microglial cells in a quiescent state, which is important for the development of memories in the hippocampus. Furthermore, focusing on the molecules involved in the CX3CL1/CX3CR1 signaling pathway could promote the creation of innovative therapeutic approaches for AD [157].

As noted earlier, CX3CL1 not only serves as an adhesion molecule but also exists in a soluble variant known as sCX3CL1. Iemmolo et al. [158] noted a reduction in CX3CL1 expression in immunofluorescence assays conducted on co-cultures of neurons, astrocytes, and microglia treated with CSF from individuals with AD. Additionally, this reduction was observed to a lesser degree in the CSF from non-AD participants when compared to the control group. This effect can be attributed to the generation of soluble CX3CL1 (sCX3CL1) by proteolytic enzymes present in the CSF [158]. Xu et al. [159] revealed that the concentrations of urinary CX3CL1 increase with age and are positively associated with aging. In contrast, individuals diagnosed with Alzheimer's disease exhibited markedly higher levels of urinary CX3CL1 when compared to cognitively normal controls and those with amnestic mild cognitive impairment (aMCI). Therefore, urinary CX3CL1 levels are linked to the aging process and could potentially act as a diagnostic biomarker for both aMCI and AD.

Existing evidence indicates that the abnormal expression of CX3CL1 in AD and its association with neuroinflammation and synaptic damage underscore its potential as a biomarker. However, to fully establish its value, a single study must meet several conditions: longitudinal cohort validation, which necessitates the verification of the correlation between CX3CL1 levels and the progression of AD in a large sample cohort; and specificity analysis, which requires the exclusion of interference from other neurodegenerative diseases or inflammatory states.

### 8.6. miRNA

RNA, as a crucial molecule in protein synthesis, significantly reflects the expression status of proteins. Among the different varieties of RNA, ribosomal RNA (rRNA) and transfer RNA (tRNA) are the most commonly found. However, with ongoing research advancements, a growing number of non-coding RNA types have been discovered, including endogenous small non-coding RNAs (miRNAs) and other non-coding RNAs (ncRNAs). In the pathogenesis of AD, the function of microRNA (miRNA) is especially crucial. Researchers, including Chai et al. [160], have explored the potential application of miRNA isolated from extracellular vesicles (EVs) in peripheral blood samples for the non-invasive diagnosis of AD. They conducted a comparative analysis of miRNAs extracted from EVs and serum in patients with AD versus a control group, examining the associations between these miRNAs and AD. The study's findings indicated that, unlike serum miRNAs, EV-derived miRNAs associated with AD exhibited more significant differences. Duan et al. [161] gathered data from a cohort comprising 71 patients with AD and 71 healthy individuals. The findings indicated that the serum-derived exosomes from AD patients exhibited differential expression of several microRNAs, including hsa-miR-125b-1-3p, hsa-miR-193a-5p, hsa-miR-378a-3p, hsa-miR-378i, and hsa-miR-451a, when compared to those of healthy subjects [161]. The ROC analysis revealed that hsa-miR-125b-1-3p achieved an AUC of 0.765 in the AD cohort, showcasing a sensitivity of 82.1% and a specificity of 67.7% [161]. Furthermore, hsa-miR-451a presented an AUC of 0.728, successfully distinguishing between the AD and healthy groups, with a sensitivity of 67.9% and a specificity of 72.6% [161]. Liu et al. [162] identified that the level of miR-24-3p was elevated by 1.6 times in the AD cohort (n=104) in comparison to the healthy control group (n=94). Furthermore, a negative relationship was noted between the levels of miR-24-3p and the MMSE scores. The ROC curve revealed adequate diagnostic accuracy. Lie et al. [163] patients diagnosed with AD show increased levels of ABCA1 in both serum and CSF exosomes. Furthermore, the concentrations of exosomal miR-193b associated with ABCA1 are similarly heightened in the CSF and serum of individuals with AD [163]. This highlights the promise of EVs as tools for identifying prognostic biomarkers associated with neurodegenerative diseases, simultaneously offering important insights for creating novel diagnostic biomarkers for AD.

### 8.7. Imaging Biomarkers

In the realm of diagnosing and researching AD, imaging biomarkers play a crucial role. The Aβ positron emission tomography (Aβ-PET) technique provides the significant advantage of directly visualizing the deposition of Aβplaques within the brain. This capability renders Aβ-PET as a valuable tool for the early detection and screening of AD [164-166]. By allowing the identification of plaques that represent key aspects of AD pathology, Aβ-PET improves our comprehension of the progression of the disease and supports prompt intervention strategies for those impacted. Tau positron emission tomography (Tau-PET) emphasizes the buildup of tau protein in the brain, utilizing advanced imaging techniques that distinctly illustrate the irregular clustering of tau proteins. This technique holds significant value for a comprehensive understanding of disease progression and for the accurate assessment of disease severity [167-169]. Fluorodeoxyglucose Positron Emission Tomography (FDG-PET) serves as an indirect measure of neuronal function by monitoring levels of cerebral glucose metabolism. A decrease in glucose metabolism within the brain suggests impaired neuronal activity [170, 171]. MRI accurately measures the volume of the hippocampus, thus serving as a valuable tool for diagnosing, evaluating disease progression, and tracking treatment in AD [172, 173].

## 9. Discussion

In the past decades, significant advancements have been made in the study of biomarkers for AD, covering numerous aspects including Aβ accumulation, tau-related pathology, neurodegenerative processes, and inflammatory responses [174]. Biomarkers are vital for the diagnosis and prediction of AD, functioning as key instruments for the early identification and management of this disorder. Clinically, AD is characterized by its insidious onset, often resulting in late detection, and the management of disease symptoms becomes increasingly challenging post-diagnosis. Consequently, achieving early detection and diagnosis would substantially enhance patient treatment outcomes. This article reviews both core and novel biomarkers associated with AD. The core value of AD blood biomarkers lies in their non-invasiveness, accessibility, and potential for dynamic monitoring. Firstly, the non-invasive nature of sample collection makes it applicable to a wider population, allowing for large-scale community screening (such as health check-ups and epidemiological surveys). Furthermore, the transport and storage of blood samples are made easier by their stability, allowing them to stay stable at room temperature for multiple hours. In contrast, CSF requires immediate centrifugation and must be kept at -80°C, as biomarkers like Aβ and tau are susceptible to degradation or changes in conformation. The feasibility of dynamic monitoring is strong, enabling tracking of changes in biomarker concentrations, whereas CSF is difficult to collect repeatedly. The timeliness of diagnosis was improved, as blood testing can yield results in 15 to 30 minutes, making it suitable for rapid triage in emergency or memory clinics; in contrast, CSF testing relies on central laboratories and typically takes 1 to 3 days. Blood tests can simultaneously detect multiple biomarkers, while CSF testing is limited by sample volume, necessitating batch testing for multi-biomarker detection, which increased the risk of error. Furthermore, the cost of simultaneous testing is significantly reduced, eliminating the need for anesthesia and nursing costs associated with lumbar punctures (Table 1).

**Table 1:** AD Biomarker Detection Methods and Clinical Significance.

Marker Type	Specific indicators	Testing method		Clinical significance	Ref.
Cerebrospinal fluid biomarkers	Aβ42	ELISA, ECLIA, CLEIA, Simoa, Mass spectrometry (MS), etc		A decrease in Aβ42 levels reflects Aβ plaque deposition	[34-36]
	Aβ42/Aβ40 ratio	Lumipulse G β-Amyloid Ratio		Used for AD diagnosis and highly consistent with Aβ-PET results.	[37-43
	Phosphorylated tau protein (P-tau)	ELISA, ECLIA, CLEIA, Simoa, Mass spectrometry (MS), etc		The rise in p-tau levels indicates the development of NFTs within the brain.	[47-48]
Blood biomarkers	Aβ42/Aβ40	SIMOA, ELISA, IP-MS		A decrease in the Aβ42/Aβ40 ratio better reflects reduced Aβ42 clearance and plaque deposition in the brain.	[64-69]
	p-tau181	Simoa® phospho-tau 181 (p-tau181) Blood test kit		Elevated levels of p-tau181 in the blood are associated with AD pathology.	[79-80]
	p-tau217	Simoa® phospho-tau 217 (p-tau217) Blood test kit		Increased concentrations of blood p-tau217 indicate the development of NFTs within the brain.	[81-85]
	p-tau231	SIMOA, Mass spectrometry (MS), etc		In AD, increased concentrations of blood p-tau231 are directly linked to the development of NFTs.	[87-88]
	p-tau205	ELISA, Simoa		Its elevation may predate Aβ deposition, suggesting initiation of Tau pathology, which is positively correlated with load of NFTs.	[89-90]
Research on other body fluid biomarkers	Saliva	Saliva testing		Salivary levels of total tau protein (t-tau) and phosphorylated tau protein (p-tau) may indicate the tau pathology associated with AD. Additionally, lactoferrin present in saliva might act as a potential biomarker for AD.	[94-96]
	Urine	Urine test		AD7c-NTP (Neurofilament Protein) reflects neuronal damage. Formaldehyde (FA) in urine reflects oxidative damage. The combination of urine biomarkers enables efficient diagnosis of AD and achieves non-invasive detection.	[98-104]
	Tear fluid	Tear fluid test		Decreased tear secretion is correlated with cognitive impairment; eIF4E and miRNA in tears may reflect AD pathology.	[106-109]
Novel biomarker	Neurofilament Light Chain (NfL)	SIMOA, Mass spectrometry (MS)		Elevated NfL reflects axonal damage.	[111-116]
	Neurogranin (Ng)	ELISA,Mass spectrometry (MS)		Elevated CSF Ng levels reflect synaptic dysfunction and synaptic loss.	[118-121]
	β-secretase 1 (BACE1)	ELISA		Elevated BACE1 activity is closely associated with Aβ plaque deposition in AD.	[124-128]
	Synaptosome-associated protein 25 (SNAP-25).	Cerebrospinal fluid/blood test		Synaptic dysfunction markers, reflecting neuronal connection damage.	[132-134]
	Inflammation-related factors	Cytokines	Cerebrospinal fluid/blood test	Inflammatory factors (IL-6/8/23/33, TNF-α) are significantly elevated in the blood of AD patients.	[136-141]
	GFAP			Increased GFAP levels predict AD pathology and treatment response	[142-145]
	YKL-40			Elevated YKL-40 levels reflect AD neuroinflammation and Aβ pathological activity	[146-147]
	CX3CL1			CX3CL1 influences AD progression by regulating neuroinflammation	[148-159]
	miRNA	Cerebrospinal fluid/blood test		MicroRNAs possess considerable promise in the disease mechanisms, assessment, and therapy of AD.	[160-163]
Imaging Biomarker	Aβ-PET	Positron Emission Tomography		Directly visualize Aβplaque deposition in the brain.	[164-166]
	Tau-PET	Positron Emission Tomography		Directly visualize tau protein deposition in the brain.	[167-169]
	FDG-PET	Positron Emission Tomography		Shows decreased cerebral glucose metabolism, reflecting neuronal dysfunction.	[170-171]
	Hippocampal volume (MRI).	Magnetic Resonance Imaging (MRI).		Reduction in hippocampal volume reflects brain atrophy.	[172-173]

However, there are several issues that need to be addressed for the in-depth promotion of blood biomarkers: 1) Lack of detection standards and insufficient validation: The detection methods for blood biomarkers (such as ELISA and mass spectrometry) have not yet established a unified industry standard, resulting in significant differences in sensitivity among kits from different manufacturers, which leads to poor comparability of clinical data. 2) Incomplete clinical evidence chain: There is a lack of large-scale, multi-center validation studies for blood biomarkers. 3) Ethical controversies regarding screening in asymptomatic populations: Although the non-invasive nature of blood testing may promote the extension of AD screening to healthy populations, the diagnosis of Aβ positivity (preclinical AD) may induce psychological anxiety and poses ethical risks of 'overdiagnosis'. 4) Difficulty in implementing grassroots medical technology: This limits the accessibility of blood biomarkers. When extracting plasma biomarkers, it is crucial to consider comorbidities and individual differences. Chronic kidney disease can nonspecifically alter the concentration of plasma biomarkers by reducing the glomerular filtration rate, primarily affecting NfL and GFAP. A high body mass index significantly influences the levels of various plasma biomarkers, with the most pronounced effect observed on NfL. Additionally, hypercholesterolemia can enhance the association between dementia risk and plasma biomarkers. The permeability of the BBB varies in AD due to disease stage and individual factors, which can affect the transport of biomarkers from the CNS to the periphery, thereby influencing the detection of plasma biomarkers. This is particularly evident in the case of plasma Aβ, as the high viscosity of Aβ in plasma complicates its transport across the BBB to the periphery. Furthermore, various proteins and cells in the blood can bind to Aβ, masking its antigenic epitopes and making it harder for antibodies to capture it. Additionally, factors such as ethnicity, *APOEε4* genotype, age, sex, and medication can also influence the measurement of plasma biomarkers.

While considerable advancements have been made in the study of AD biomarkers recently, several challenges remain to be addressed. In the future, it is crucial to continually enhance the sensitivity and specificity of detection technologies to accurately quantify low-concentration biomarkers in CSF and blood. Additional studies are essential to explore the connection among Aβ, tau protein, and neuroinflammation, thereby laying the groundwork for the development of targeted therapies. Ongoing efforts are essential to improve the application of non-invasive biomarkers in clinical settings, thus facilitating widespread screening and early disease identification. It is vital to continuously develop and accurately implement novel biomarkers for AD. Through the development of combined models utilizing multi-dimensional biomarkers, further references can be offered for the early detection of AD. As technology progresses and multi-marker joint analyses evolve, biomarkers are anticipated to assume a growingly important function in precision medicine and the early intervention of AD, offering renewed hope to those affected.

In summary, the determination of plasma biomarkers is influenced by various factors, making it essential to establish threshold values for clinical diagnosis. Currently, this area remains in its early stages, and future research should be focused on standardizing the biomarker detection process. This includes analyzing influencing factors, unifying detection methods and tools, and refining detection technologies to enhance the comparability of data across different studies. With the iterative upgrades of artificial intelligence technology, the future trend will undoubtedly be the integration of AI into the early diagnosis of diseases. Certain studies have already utilized machine learning algorithms to perform cross-modal feature fusion between biomarkers and imaging data, constructing high-precision predictive models. This advancement not only enhances diagnostic accuracy but also provides a technological bridge for the development of precision medicine.

## Data Availability

All of the data used to support the findings of this study are included within the article.
